# Hormone References for Ultrasound Breast Staging and Endocrine Profiling to Detect Female Onset of Puberty

**DOI:** 10.1210/clinem/dgaa679

**Published:** 2020-09-22

**Authors:** Andre Madsen, Ingvild S Bruserud, Bjørn-Erik Bertelsen, Mathieu Roelants, Ninnie Helen Bakken Oehme, Kristin Viste, Robert Bjerknes, Bjørg Almås, Karen Rosendahl, Gunnar Mellgren, Jørn V Sagen, Petur B Juliusson

**Affiliations:** 1 Hormone Laboratory, Department of Medical Biochemistry and Pharmacology, Haukeland University Hospital, Bergen, Norway; 2 Department of Clinical Science, University of Bergen, Bergen, Norway; 3 Department of Pediatrics, Haukeland University Hospital, Bergen, Norway; 4 Environment and Health, Department of Public Health and Primary Care, KU Leuven, University of Leuven, Leuven, Belgium; 5 Department of Radiology, University Hospital of North Norway, Tromsø, Norway; 6 Department of Clinical Medicine, University of Tromsø, The Arctic University of Norway, Tromsø, Norway; 7 Mohn Nutrition Research Laboratory, Department of Clinical Science, University of Bergen, Bergen, Norway; 8 Department of Health Registries, Norwegian Institute of Public Health, Bergen, Norway

**Keywords:** female puberty, estrogen, pediatric endocrinology, references, hormones, principal component analysis

## Abstract

**Context:**

Application of ultrasound (US) to evaluate attainment and morphology of glandular tissue provides a new rationale for evaluating onset and progression of female puberty, but currently no hormone references complement this method. Furthermore, previous studies have not explored the predictive value of endocrine profiling to determine female puberty onset.

**Objective:**

To integrate US breast staging with hypothalamic-pituitary-gonadal hormone references and test the predictive value of an endocrine profile to determine thelarche.

**Design Setting and Participants:**

Cross-sectional sample of 601 healthy Norwegian girls, ages 6 to 16 years.

**Main Outcome Measures:**

Clinical and ultrasound breast evaluations were performed for all included girls. Blood samples were analyzed by immunoassay and ultrasensitive liquid chromatography–tandem mass spectrometry (LC-MS/MS) to quantify estradiol (E_2_) and estrone (E_1_) from the subpicomolar range.

**Results:**

References for E_2_, E_1_, luteinizing hormone, follicle-stimulating hormone, and sex hormone–binding globulin were constructed in relation to chronological age, Tanner stages, and US breast stages. An endocrine profile index score derived from principal component analysis of these analytes was a better marker of puberty onset than age or any individual hormone, with receiver-operating characteristic area under the curve 0.91 (*P* < 0.001). Ultrasound detection of nonpalpable glandular tissue in 14 out of 264 (5.3%) girls with clinically prepubertal presentation was associated with significantly higher median serum levels of E_2_ (12.5 vs 4.9 pmol/L; *P* < 0.05) and a distinct endocrine profile (arbitrary units; *P* < 0.001).

**Conclusions:**

We provide the first hormone references for use with US breast staging and demonstrate the application of endocrine profiling to improve detection of female puberty onset.

A comprehensive metastudy recently established a statistically significant and still ongoing trend of earlier breast development (thelarche, ie, puberty onset) in girls, corresponding to almost 3 months per decade since 1977 (1). This finding underlines the need to compile updated references for puberty development milestones and pertinent hormones used in the diagnosis and management of altered puberty onset in the current pediatric population.

Hypothalamic activation initiates female puberty by enhanced release of pituitary gonadotropins, mainly luteinizing hormone (LH) and follicle-stimulating hormone (FSH) to stimulate gonadal maturation (2). Abnormal circulating levels of FSH and LH in conjunction with gonadal hormones can support the diagnosis of pituitary malfunction, hypogonadism and various disorders of sexual development associated with altered puberty onset (3-5). The main female sex steroids estrone (E_1_) and estradiol (E_2_) are involved in development and maintenance of the female phenotype and gonadal function. In particular, E_2_ is an integral biomarker for assessing female pubertal timing, menstrual status, and fertility.

Female puberty onset is traditionally defined by breast formation, specifically, attainment of palpable glandular breast tissue and areolar enlargement, corresponding to Tanner stage B2 (6). In contrast, ultrasound (US) leverages short-wavelength echogenicity of different tissues to render visual representations of internal anatomic structures and morphology. Evaluation of breast glandular tissue by US is a new approach for assessing female puberty, but its clinical utility remains unexplored and compatible hormone references have been lacking. Notably, formation of glandular tissue in early thelarche may be detectable by US but not palpable by hand (7). In the pediatric subspecialist setting, US breast staging may improve clinical investigations of altered puberty timing since this method allows for storage and objective retrospective assessments of digital images during longitudinal patient follow-up. Moreover, the procedure is harmless (8, 9); perceived by the patient as less invasive than palpation (our unpublished observations); and provides better definition of breast maturation than Tanner stages (10), while being able to differentiate between glandular and adipose tissue in overweight girls where excess fat accretion may confound traditional breast staging by palpation and visual inspection (11, 12). Age references for both breast staging by clinical Tanner and US breast examinations are provided (Fig. 1) in line with our previously published population study (7).

**Figure 1.**
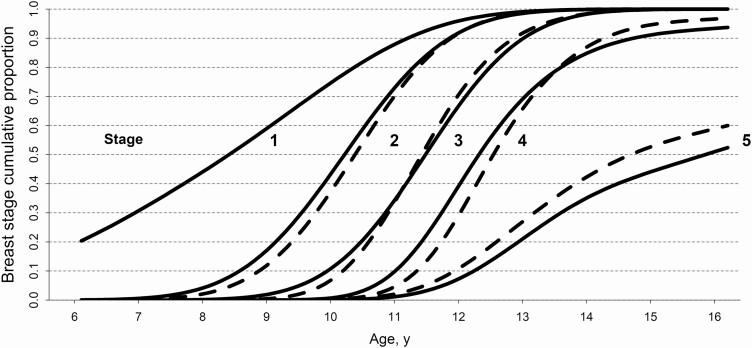
Age reference curves for female puberty. Probit models for age of occurrence for indicated breast stages determined clinically (Tanner B; dashed lines) and by ultrasound (US B; solid lines). The US B1 stage was defined by prepubertal breast morphology, but it was radiologically distinct and more advanced than the baseline stage US B0.

In medicine and biology, principal component analysis (PCA) is a statistical approach to stratify patients or identify phenotype clusters by capturing the variance from several variables, or dimensions (13). Previous applications of PCA in pediatric research include composite risk index scoring for metabolic syndrome (14) and identification of allergy phenotypes (15). Endocrine profiling by PCA was previously demonstrated to identify distinct subtypes of Cushing syndrome (16), improve the predictive value of newborn screening for congenital adrenal hyperplasia (17), and extract endocrine phenotypes with implications for puberty timing in a longitudinal study of female puberty (18). However, the utility of a reference endocrine profile as diagnostic marker of puberty onset remains unexplored.

From the female pediatric population sample in the cross-sectional Bergen Growth Study 2 (BGS2) in Norway, we aimed to establish hormone references in relation to both traditional Tanner and US breast stages. We leveraged a newly established in-house liquid chromatography–tandem mass spectrometry (LC-MS/MS) method to quantify estrogens in the subpicomolar range (19) in order to investigate whether US stratification of clinically prepubertal girls would reveal biochemically distinct phenotypes. Lastly, we explored the predictive value of hormone biomarkers and an endocrine profile to determine female puberty onset.

## Materials and Methods

### Cohort description

Clinical examinations and data collection for the BGS2 cross-sectional cohort was conducted in 2016. Children in the age interval from 6 to 16 years from 6 schools were voluntarily recruited with parental consent to be examined regarding puberty status. This cohort was described previously (7). Briefly, a total of 703 girls were included in the cohort (participation rate 49.5%), of whom 651 donated blood samples. The following participants were excluded: 27 due to chronic disease; 12 due to use of oral contraceptives, and 11 due to insufficient blood sample volume.

### Clinical and ultrasound evaluation of puberty

Breast maturation was evaluated both clinically (Tanner B) and with US. US images were analyzed after study completion to prevent observer bias. Clinical evaluation was performed in accordance with the schematics proposed by Marshall and Tanner (6) and included breast palpation. An experienced radiologist established the US staging protocol in line with a previous study (20). Description of the morphological distinctions providing the basis for US breast staging was provided previously (21). A trained nurse performed all clinical and US evaluations, and the most mature breast was examined. Briefly, stage US B0 was characterized as a small hypoechoic area in the retro-areolar area. Unique to stage US B1 was the presence of hyperechoic tissue with a triangular shape. In stage US B2, corresponding to clinical thelarche, the internal breast was characterized by a small hypoechoic center (linear, round, or star-shaped) with surrounding glandular hyperechoic tissue. The presence of a hypoechoic center was a prerequisite for stages US B3 and US B4, and while the appearance in US B3 was spider-shaped, the center was defined as increasingly roundish in US B4. The mature stage US B5 presented as a heterogeneous mass devoid of the hypoechoic center. As described previously (21), the intra-observer agreement of US B staging was “very good” (Cohen’s kappa 0.84; 95% CI, 0.78-0.91). Presence of pubic hair was defined as a score of 2 or higher according to the Tanner PH scale (22).

### Blood sample analyses

Samples were collected from venous blood between 8:20 am and 2:10 pm. Average time of blood draw was 10:57 am and cumulative proportion of samples collected according to time of day starting from 08:20 is provided: 09:00 am (9%); 10:00 am (30%); 11:00 am (53%); 12:00 pm (69%); 1:00 pm (88%); 2:00 pm (99%); 2:10 pm (100%). Serum was stored at −80 °C prior to analysis at the Hormone Laboratory, Department Of Medical Biochemistry and Pharmacology, Haukeland University Hospital Hormone Laboratory, where personnel were blinded for participant age and pubertal status. The laboratory and its analytical practice are accredited in accordance with NS-EN ISO 15189:2012. The following coefficients of variation (CV) refer to the inter-assay variation. Siemens IMMULITE 2000 XPi was used to analyze basal levels of LH (CV 7% at 10 IU/L), FSH (CV 5% at 17 IU/L) and sex hormone–binding globulin (SHBG; CV 6% at 6.74 µg/mL). E_2_ and E_1_ were analyzed by an ultrasensitive LC-MS/MS method as recently described (23). Briefly, samples were subjected to liquid-liquid extraction before analysis and quantification by LC-MS/MS. Lower limit of detection (LOD) was 0.28 pmol/L for E_2_ and 0.15 pmol/L for E_1_. Lower limit of quantification (LOQ) was 0.58 pmol/L for E_2_ (CV 9.1% in the range 1.7-153.3 pmol/L) and 0.25 pmol/L for E_1_ (CV 7.8%; range 1.7-143.1 pmol/L). To convert estradiol (E_2_) to pg/mL, divide by 3.671. To convert estrone (E_1_) to pg/mL, divide by 3.698. To convert SHBG to µg/mL, divide by 8.896.

### Hormone reference intervals

Nonparametric reference intervals were established in accordance Clinical & Laboratory Standards Institute (CLSI) EP28-A3c guidelines (24) and the Canadian Laboratory Initiative on Pediatric Reference Intervals (CALIPER) framework (25). Briefly, the central 95% range was defined by the lower 2.5th and upper 97.5th percentile limits. Reference intervals were established from breast stage partitions by resampling to 500 bootstrapped data points using the Analyse-it (Analyse-it, Leeds, UK) extension in Microsoft Excel (Microsoft, Redmond, WA, USA). The Harris-Boyd standard deviate test (26-28) was applied to log-transformed data with approximate Gaussian distributions to determine statistically significant differences between pairwise, successive breast stage partitions. Justified partitioning criteria were met if the numeric Harris-Boyd z-score exceeded that of the critical z* sample power test, as mathematically outlined previously (26-29). Continuous hormone reference intervals in relation to chronological age were generated using the CLSI-compliant *referenceIntervals* package in *R* (R Development Core Team, Vienna, Austria), based on a moving window of 120 observations as described previously (29).

### Endocrine profiling

Data dimensionality reduction by PCA was applied to generate a composite endocrine profile score for serum level constellations of hormones E_1_, E_2_, LH, FSH, and SHBG. PCA was applied to hormone data from 403 premenarcheal girls (age interval, 6.2-15.7 years). Participants with missing data for one or more hormones were discarded. Postmenarcheal girls were excluded from PCA in order to omit menstrual cycle hormone fluctuations. The first principal component 1 (PC1) comprised the following loadings: E_1_ (0.497), E_2_ (0.494), LH (0.475), FSH (0.469), and SHBG (−0.252). This PC1 exhibited an Eigenvalue of 3.6 (ie, its standard deviation of 1.9 squared) and accounted for 70.0% of the hormone dataset variance. Secondary PCs returned Eigenvalues below 1.0 and were accordingly discarded. Participant PC1 scores were thus used to assign individual endocrine profiles in context of the total dataset variance. The PCA was computed in *R* with code operations provided as supplemental data (30).

### Ethical considerations

The BGS2 was approved by the Norwegian Regional Ethics Committee, case references 2015/128/REK and 2015/235/REK. The study design and conduct conformed to good clinical practice and the ethical decrees of the Helsinki Declaration. Children younger than 16 years were only examined with written and informed parental consent and child assent, both of which were instantly revocable. Participants were rewarded with a cinema voucher. Breast palpation or US performed in the study did not detect any pathology.

### Statistical analyses

Transition to pubertal breast stage was estimated from age using probit regression for a generalized linear model in *R,* as described previously (7). Receiver-operating characteristics (ROC) curves were generated using *R* and the optimal ROC cutoff value (Youden index) was computed using with the *pROC* package, corresponding to the point of the ROC curve where the sum of sensitivity and specificity for distinguishing 2 groups is highest (31). Statistical significance was defined as: * *P* < 0.05, ^**^P < 0.01, ^***^P < 0.001 (2-tailed Mann-Whitney U test) and ^a^ z > z* (Harris-Boyd test).

## Results

### Hormone references in relation to chronological age

**Figure 2.**
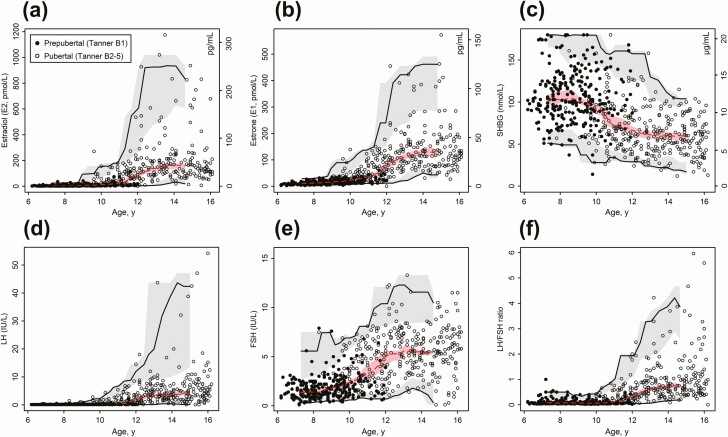
Hormone references in relation to chronological age. Age references for (a) estradiol (E_2_; No. = 547), (b) estrone (E_1_; No. = 561), (c) luteinizing hormone (LH; No. = 600), (d) follicle-stimulating hormone (FSH; No. = 599), (e) sex hormone–binding globulin (SHBG; No. = 601) and (f) LH/FSH ratio (No. = 599). All healthy participants were included, and the variable number of observations for the individual hormones were due to insufficient serum volume to determine the respective analytes. Filled dots represent prepubertal girls (Tanner B1) and open dots represent pubertal girls (Tanner B2+). Continuous median, lower limit (2.5th percentile) and upper limit (97.5th percentile) centiles with 90% CIs were estimated by nonparametric method from a moving window of 120 observations.

Serum levels of E_2_, E_1_, LH, FSH, SHBG, and LH/FSH ratio were plotted against age and reference centiles defining the 95% normal range and median were computed from a moving window of 120 observations (Fig. 2). Table format hormone references interpolated from these models are provided in SI units in Supplemental Table 1 (32) and conventional units in Supplemental Table 2 (33).

### Hormones reference intervals by puberty stages

Reference intervals according to breast evaluations by clinical examination (Tanner B stages) and US (US B stages) were established by bootstrapping breast stage partitions to 500 observations (Table 1). The equivalent table with analytes annotated in conventional units is provided as Supplemental Table 3 (34). Justified partitioning of incremental breast stage reference intervals was verified using the pairwise Harris-Boyd standard deviate test (Z). Where indicated not significant (*n.s.*), the reference interval overlap of the current and the previous partition was too extensive to warrant partitioning despite the dichotomized segregation by breast stages. By the same logic, standard deviate testing of corresponding Tanner B and US B stage reference intervals (ie, Tanner B2 vs US B2, with matching increments) showed agreement between the 2 methods with no statistical evidence to support endocrinological distinctions for any of the 5 hormones. Median ages (95% CI) for the Tanner B stages were: B1, 8.7 (6.6-11.6); B2, 10.6 (8.5-13.8); B3, 11.9 (10.2-15.1); B4, 14.0 (11.6-16.0), and B5, 14.6 (11.7-16.0) years. Median ages for the US B stages were: US B0, 8.2 (6.5-11.4); US B1, 8.9 (6.8-11.9); US B2, 10.6 (8.3-13.8); US B3, 12.0 (9.6-15.8); US B4, 13.8 (11.2-16.0), and US B5, 14.4 (12.1-16.0) years.

**Table 1.** Reference Intervals for Tanner and US Breast Stages

				95% Reference intervals		
**Reference**	**Stage**	**No.**	** Median**	**2.5th percentile (90% CI)**	**97.5th percentile** (**90% CI)**	**Z**
Estradiol (E_2_), pmol/L LC-MS/MS						
Tanner stage	B1	260	4.8	0.9 (0.7-1.1)	38.0 (22.9-65.2)	-
	B2	69	24.0	6.0 (4.9-6.9)	204.4 (120-291)	a
	B3	63	90.0	4.3 (0.7-10.6)	280.6 (221-302)	a
	B4	73	177	26.3 (7.2-56.5)	803.4 (634-1020)	a
	B5	76	150	48.4 (42.0-65.0)	981.5 (838-1175)	n.s.
Ultrasound stage	US B0	134	4.4	0.8 (0.7-1.1)	38.8 (20.3-61.4)	-
	US B1	111	5.1	1.2 (0.7-1.7)	42.6 (20.0-71.0)	n.s.
	US B2	78	23.5	3.7 (2.3-6.0)	203.3 (148-302)	a
	US B3	60	108	6.0 (3.3-10.4)	453.3 (303-536)	a
	US B4	90	151	19.9 (0.7-58.3)	834.3 (662-1020)	a
	US B5	63	160	30.2 (7.2-55.8)	1012.7 (779-1175)	n.s.
Estrone (E_1_), pmol/L LC-MS/MS						
Tanner stage	B1	260	14.0	4.4 (3.6-5.0)	37.9 (34.0-45.0)	-
	B2	69	34.0	12.6 (12.0-14.5)	119.1 (96.3-139)	a
	B3	66	78.5	12.3 (0.4-31.0)	192.4 (151-214)	a
	B4	78	118	41.8 (27.0-53.0)	398.9 (290-423)	a
	B5	81	135	55.2 (50.0-63.0)	476.1 (385-573)	a
Ultrasound stage	US B0	134	13.0	3.9 (2.7-4.5)	38.8 (33.8-47.8)	-
	US B1	111	16.0	5.6 (3.1-6.3)	41.9 (34.0-75.0)	a
	US B2	78	32.5	11.2 (7.3-13.8)	154.5 (100-214)	a
	US B3	62	80.5	17.1 (14.9-26.3)	192.0 (177-199)	a
	US B4	97	115	33.4 (0.4-53)	426.9 (337-573)	a
	US B5	67	135	43.4 (27.0-57.8)	435.9 (387-463)	a
LH, IU/L IMMULITE 2000 XPi						
Tanner stage	B1	269	0.1	≤ 0.1	0.5 (0.4-0.6)	-
	B2	73	0.3	≤ 0.1	3.9 (2.3-5.4)	a
	B3	71	2.7	≤ 0.1	8.6 (6.8-9.5)	a
	B4	90	3.6	0.2 (0.1-0.6)	39.6 (16.5-54.2)	a
	B5	89	4.6	0.7 (0.4-1.0)	34.8 (17.3-47.1)	n.s.
Ultrasound stage	US B0	140	0.1	≤ 0.1	0.4 (0.3-0.5)	-
	US B1	114	0.1	≤ 0.1	0.4 (0.4-1.0)	n.s.
	US B2	83	0.2	≤ 0.1	3.6 (2.2-5.4)	a
	US B3	65	2.8	0.1 (0.1-0.2)	12.2 (8.7-16.9)	a
	US B4	111	4.3	0.2 (0.1-0.5)	37.6 (15.2-54.2)	a
	US B5	74	4.1	0.4 (0.1-0.9)	34.7 (18.3-47.1)	n.s.
FSH, IU/L IMMULITE 2000 XPi						
Tanner stage	B1	267	1.6	0.4 (0.4-0.5)	5.3 (4.6-6.4)	-
	B2	73	3.3	1.3 (1.2-1.3)	9.1 (7.4-12.1)	a
	B3	71	5.4	1.3 (1.1-1.6)	10.6 (8.4-12.3)	a
	B4	90	5.5	0.4 (0.1-1.2)	11.1 (9.5-13.3)	n.s.
	B5	90	6.1	1.4 (1.1-1.9)	10.9 (10.0-11.5)	n.s.
Ultrasound stage	US B0	138	1.6	0.4 (0.3-0.5)	5.1 (3.5-7.2)	-
	US B1	114	1.5	0.4 (0.1-0.6)	5.0 (4.5-5.6)	n.s.
	US B2	83	3.2	1.2 (1.1-1.3)	7.8 (7.3-8.1)	a
	US B3	65	5.6	0.7 (0.1-1.5)	10.6 (8.5-12.3)	a
	US B4	112	5.6	1.2 (0.1-2.1)	11.4 (8.7-13.3)	n.s.
	US B5	74	6.1	1.0 (0.3-1.7)	11.0 (10.1-11.5)	n.s.
SHBG, nmol/L IMMULITE 2000 XPi						
Tanner stage	B1	269	102	51.5 (46.0-56.8)	177.7 (≥ 174)	-
	B2	73	78.0	35.6 (30.0-41.6)	164.2 (≥ 137)	a
	B3	71	62.0	28.1 (26.0-32.8)	126.3 (114-130)	a
	B4	90	60.5	27.9 (24.0-32.6)	110.9 (101-121)	a
	B5	90	54.0	15.5 (12.0-20.3)	99.8 (90.1-105)	a
Ultrasound stage	US B0	140	103	56.0 (51.0-64.0)	179.4 (≥ 176)	-
	US B1	114	99.5	50.4 (37.0-57.9)	169.2 (≥ 155)	a
	US B2	83	83.0	28.5 (14.0-39.2)	158.6 (≥ 130)	a
	US B3	65	62.0	27.0 (25.0-33.2)	130.4 (102-158)	a
	US B4	112	57.0	19.3 (12.0-26.5)	119.0 (104-126)	a
	US B5	74	56.5	19.8 (17.0-26.0)	102 (92.5-112)	n.s.

Sample size (n) and analyte levels corresponding to the median and resampled 95% reference intervals are presented for indicated puberty breast stage partitions. Column “Z” summarizes Harris-Boyd standard deviate tests to determine justified partitioning of current and the previous partition (^a^yes) or not (n.s., not significant).

Abbreviations: B, Tanner breast stage; E_1_, estrone; E_2_, estradiol; FSH, follicle-stimulating hormone; LH, luteinizing hormone; SHBG, sex hormone–binding globulin; US B, ultrasound breast stage.

### Endocrine profile detects thelarche

Next, we evaluated the predictive value of age, endocrine profile, and individual analytes, respectively, in determining the transition to puberty onset defined by attainment of breast stage Tanner B2+ or US B2+ (Table 2). For these analyses, we selected the age interval 8.0 to 12.0 years that defined both the earliest and latest occurrence of Tanner B2 in the dataset. Statistically significant differences were observed for all variables between prepubertal and pubertal groups (*P* < 0.001 for all, Mann-Whitney U). Notably, the endocrine profile index (ie, participant PC1 scores) returned the highest area under the ROC curve and best negative predictive value (NPV) to distinguish prepubertal and pubertal girls.

**Table 2.** Predictive Markers of Female Puberty Onset

	Girls age 8-12 y	Tanner B1	Tanner B2+	Receiver-operating characteristics (ROC) curve output					
				AUC	PPV (%)	NPV (%)	Cutoff	Sens.	Spec.
**Tanner B**	Sample size, No.	185	116						
	Age, y	9.3 (8.0-11.8)	10.5 (8.6-11.9)	0.85	71.4	85.1	10.2 y	0.78	0.81
	Endocrine profile, AU	−1.0 (–1.6 to 0.5)	0.1 (−1.1 to 2.6)	0.91	75.0	88.4	-0.5 AU	0.80	0.85
	Estradiol E_2_, pmol/L	6.2 (1.0-40.7)	24.0 (6.4-126)	0.90	84.8	84.1	19.5 pmol/L	0.72	0.92
	Estrone E_1_, pmol/L	17.0 (5.1-39.1)	33.0 (13.5-98)	0.84	77.8	86.0	28.5 pmol/L	0.77	0.87
	LH, IU/L	0.1 (≤ 0.5)	0.2 (0.1-2.4)	0.84	82.8	81.2	0.2 IU/L	0.66	0.91
	FSH, IU/L	1.7 (0.6-5.1)	3.3 (1.3-7.5)	0.82	67.9	84.0	2.6 IU/L	0.76	0.77
	SHBG, nmol/L	102 (52-179)	75 (37-159)	0.80	66.9	82.2	86.5 nmol/L	0.73	0.77
	**Girls age 8–12 y**	**US B0/1**	**US B2+**	**AUC**	**PPV (%)**	**NPV (%)**	**Cutoff**	**Sens.**	**Spec.**
**Ultrasound**	Sample size, No.	170	126						
	Age, y	9.3 (8.0-11.9)	10.8 (8.5-12.0)	0.81	68.2	83.1	9.9 y	0.81	0.72
	Endocrine profile, AU	−1.0 (−1.6 to 0.45)	0.4 (−1.1 to 4.8)	0.90	71.9	88.8	-0.7 AU	0.85	0.78
	Estradiol E_2_, pmol/L	6.3 (1.0-41.6)	35.0 (3.3-242)	0.87	86.8	79.1	19.5 pmol/L	0.66	0.92
	Estrone E_1_, pmol/L	17.0 (4.7-39.9)	43.0 (12.0-139)	0.84	79.4	80.6	28.5 pmol/L	0.71	0.87
	LH, IU/L	0.1 (≤ 0.4)	0.4 (0.1-6.7)	0.81	86.8	77.1	0.3 IU/L	0.63	0.93
	FSH, IU/L	1.7 (0.5-5.0)	3.8 (1.3-8.1)	0.82	70.8	79.4	2.6 IU/L	0.73	0.78
	SHBG, nmol/L	102 (54-180)	70 (30-133)	0.79	71.7	77.3	84.5 nmol/L	0.68	0.80

Baseline characteristics (median, 95% CI) for girls grouped by Tanner or US definitions of puberty onset in the thelarche age window 8 to 12 years were leveraged in receiver-operating characteristics (ROC) analyses. Sens. (sensitivity) and Spec. (specificity) represent ROC curve coordinates for the optimal cutoff point.

Abbreviations: AU, arbitrary units; AUC, area under the curve; NPV, negative predictive value; PPV, positive predictive value.

### Ultrasound evaluation allows for refined characterization of early thelarche

Minor disagreements between Tanner and US breast staging were encountered on the intra-individual level, and we next decided to investigate the possible endocrine implications of these discrepancies. Stratifying each of the established Tanner B stages by the corresponding breast stages obtained by US, we observed no biochemical differences between US B strata in pubertal girls (Tanner B2-5). However, statistically significant endocrine discrepancies were evident upon stratification of clinically prepubertal Tanner B1 girls by US B stages (Table 3). Among these 264 prepubertal girls, individually evaluated as Tanner B1, US breast morphology was radiologically discernable by US as mainly prepubertal US B0 (139/264 = 52.7%) or US B1 (111/264 = 42.0%), but pubertal attainment of glandular tissue was also evident in a subset of US B2 girls (14/264 = 5.3%). Notably, both age and serum levels of E_1_ were significantly higher in US B1 relative to US B0 (*P* < 0.05 and *P* < 0.01, respectively, Mann-Whitney U). Comparing the US B1 and US B2 strata by Mann-Whitney U tests, we observed statistically significant differences in serum levels of E_2_ (*P* < 0.05), FSH (*P* < 0.01) and SHBG (*P* < 0.05), corresponding to a highly distinct endocrine phenotype indicated by the endocrine profile (*P* < 0.001). Importantly, these endocrine differences were observed despite no significant age difference between the US B1 and US B2 subgroups. In this comparison, the endocrine profile emerged as the most statistically significant variable.

**Table 3.** Baseline Characteristics for Clinically Prepubertal Girls Stratified by Ultrasound Breast Stages

	Clinical Tanner B1 (prepubertal)		
Variable	US B0	US B1	US B2
Stratum size, No.	139	111	14
Age, years	8.2 (6.5–11.4)	8.9* (6.8–11.9)	9.7 (8.0–11.6)
Pubic hair (PH2+), %	6.6%	5.4%	40.0%
Endocrine profile, AU	−1.1 (−1.7 to 0.1)	−1.1 (−1.5 to 0.0)	−0.6*** (−1.2 to 1.8)
Estradiol E_2_, pmol/L	4.4 (0.8-30.6)	4.90 (1.4-21.3)	12.5* (2.3-112.0)
Estrone E_1_, pmol/L	13.0 (4.2-35.7)	16.0** (5.4-34.0)	20.5 (7.3-60.0)
LH, IU/L	0.1 (0.1-0.4)	0.1 (0.1-0.4)	0.1 (0.1-2.1)
FSH, IU/L	1.6 (0.4-4.5)	1.5 (0.5-4.9)	2.4** (1.1-7.9)
SHBG, nmol/L	103 (56-180)	101 (53-169)	87* (14-128)

Baseline characteristics (median, 95% CI) for 264 girls with a prepubertal clinical presentation, stratified by US breast stages. Statistically significant differences (Mann-Whitney U) between pairwise strata (US B0 vs US B1, or US B1 vs US B2) are annotated.

Abbreviations: AU, arbitrary units; E_1_, estrone; E_2_, estradiol; FSH, follicle-stimulating hormone; LH, luteinizing hormone; SHBG, sex hormone–binding globulin.

Significant differences obtained by Mann-Whitney U tests between columns US B0 and US B1, or between US B1 and US B2 were denoted *P < 0.05, **P < 0.01 or ***P < 0.001.

## Discussion

In the current study, 601 healthy Norwegian girls were evaluated by both clinical and US breast evaluations, allowing for exceptional endocrine and methodological comparisons. Overall, the girls in the current Norwegian cohort exhibited median age at occurrence of Tanner B2 at 10.4 years, US B2 at 10.2 years, and menarche at 12.7 years (7), which is comparable to other Western countries (35-38). The cohort was representative of the general Norwegian demography, having a ~90% majority Caucasian population. Agreement between the 2 breast staging methods was satisfactory, as previously described (7, 21). Interestingly, our participants perceived US as a less invasive procedure compared to palpation, and the majority (67.3%) of the girls favored the US evaluation (our unpublished observations).

Although reference intervals for US and Tanner breast stage were biochemically comparable overall, US enabled detection of glandular tissue in a subset of clinically prepubertal girls with distinct biochemical baseline characteristics. From the stratification of clinically prepubertal (Tanner B1) girls by US B stages in Table 2, we observed significant endocrine profile differences between the substrata of prepubertal (US B1) and pubertal (US B2) girls. Although the subgroup of girls with detectable glandular tissue by US (US B2) was small, this finding implies that endocrine profiling by PCA may provide more sensitivity than any singular hormone in context of detecting thelarche. Further studies are warranted to explore the use-case for tailored endocrine profiles as predictive or diagnostic markers of endocrinopathies, including hypogonadism and disorders of sexual development.

The application of endocrine profiling in the current study has limitations and warrants discussion. PCA was applied to capture the total variance of 5 hormone concentrations from 403 premenarcheal girls. The first principal component, PC1, explained 70% of the total dataset variance and thus the PC1 scores associated with each study participant were leveraged as a composite endocrine profile index. The rationale for this endocrine profile was that its constituent hormones are integral components of the pubertal hypothalamic-pituitary-gonadal signaling axis and subject to reciprocal regulation. Loading other pertinent hormone dimensions into the endocrine profile would arguably generate additional complexity and depth. The application of endocrine profiling by PCA cluster analysis was recently applied to extract distinct endocrine phenotype clusters in a longitudinal study of female puberty (18). In contrast, our approach was to generate a reference endocrine profile for normal puberty development. As shown in Table 2, the composite endocrine profile index was an excellent marker of thelarche, but in terms of ROC diagnostic performance it was only marginally better than, or practically equivalent to that of E_2_ levels alone. However, the occurrence of US thelarche morphology in clinically prepubertal girls in Table 3 was marked with a higher degree of statistical confidence by the endocrine profile than by E_2_ alone. These findings imply that endocrinological investigations of altered puberty timing in some cases may benefit from PCA profiling. Automated computation for endocrine profiling may provide added value to existing laboratory systems or clinical decision trees. In a previous study, Fugl et al observed that baseline level of LH was the better analyte variable to determine thelarche (39). Notably, this study included a sample size of only 43 girls, with a late minimum inclusion age of 9.8 years.

A central undertaking in the current work was the construction of reference intervals compatible with previously defined US breast stages (7, 20, 21). Table 1 was configured to juxtapose reference intervals for clinical Tanner B stages and US B stages. Notably, applying the Harris-Boyd standard deviate test criteria, there were no cases where hormone levels differed significantly between corresponding Tanner B and US B stages throughout puberty or when pooled observations in US B0/1 was compared to Tanner B1. This implies that these 2 ordinal methods of breast staging are principally compatible in endocrinological terms, with matching breast stages aligning biochemically despite different methodological rationales. However, US enabled stratification of girls nearing thelarche.

Our reference intervals should be interpreted with some precautions. The current references represent average hormone levels throughout the morning and early noon, and do not account for intra-day variation for gonadotropins and estrogen levels. In this regard, the diurnal rhythm for estrogen, LH and FSH secretion have been extensively studies by others (40-42). Due to logistical constraints, we were unable to obtain fasting morning samples and, in line with similar studies (25, 43-46) our references rely on sample power to squelch diurnal secretion patterns and may thus be representative for outpatient clinics. Further, we did not account for latent hormone fluctuations associated with monthly cyclicity in premenarcheal girls, and postmenarche data were not stratified by menstruation cycle phase at the time of blood draw.

In conclusion, we have provided the first set of statistically robust hormone references for US breast staging, in comparison with traditional Tanner B stages for female puberty. Our results demonstrate a high degree of agreement between the 2 methods of puberty staging, both in terms of age at stage occurrences and endocrine parameters. However, US enabled detection of nonpalpable glandular tissue in a subset of clinically prepubertal girls, and this phenotype was corroborated by a pubertal endocrine profile. Furthermore, we have demonstrated that index scores from endocrine profiling by PCA represents a useful predictive marker of puberty onset with a possible use-case in detecting pediatric endocrinopathies associated with altered puberty onset.

## Data Availability

Restrictions apply to the availability of data generated or analyzed during this study to preserve patient confidentiality or because they were used under license. The corresponding author will on request detail the restrictions and any conditions under which access to some data may be provided.

## Abbreviations

BGS2: Bergen Growth Study 2
CV: coefficient of variation
E_1_: estrone
E_2_: estradiol
FSH: follicle-stimulating hormone
LC-MS/MS: liquid chromatography–tandem mass spectrometry
LH: luteinizing hormone
PCA: principal component analysis
ROC: receiver-operating characteristic
SHBG: sex hormone–binding globulin
US: ultrasound

## Additional Information

***Disclosure Summary:*** The authors have nothing to disclose.
